# GTR/NBR/Silica Composites Performance Properties as a Function of Curing System: Sulfur versus Peroxides

**DOI:** 10.3390/ma14185345

**Published:** 2021-09-16

**Authors:** Łukasz Zedler, Xavier Colom, Javier Cañavate, Krzysztof Formela

**Affiliations:** 1 Department of Polymer Technology, Faculty of Chemistry, Gdańsk University of Technology, Gabriela Narutowicza 11/12, 80-233 Gdańsk, Poland; 2 Department of Chemical Engineering, Universitat Politècnica de Catalunya Barcelona Tech, Colom 1, Terrassa, 08222 Barcelona, Spain; xavier.colom@upc.edu (X.C.); francisco.javier.canavate@upc.edu (J.C.)

**Keywords:** recycling, ground tire rubber, composites, curing system, structure-property relationship

## Abstract

In this work, conventional sulfur and two types of organic peroxides (dicumyl peroxide (DCP) and di-(2-tert-butyl-peroxyisopropyl)-benzene (BIB)) curing systems were used to investigate the possibility for tailoring of the performance properties of GTR/NBR blends reinforced with a variable content of highly dispersive silica (0–30 phr). The curing characteristics, static mechanical and acoustical properties, swelling behavior, thermal stability, and microstructure of the prepared composites were investigated. The results show that regardless of the curing system used, increasing the content of highly dispersive silica resulted in the improvement of the mechanical properties of the studied materials. It was observed that sulfur-based systems are the best choice in terms of cross-linking efficiency determined based on torque increment and cross-link density parameters. However, further analysis of the physico-mechanical properties indicated that the cross-linking efficiency does not match the performance of specimens, and the materials obtained using organic peroxides show higher tensile properties. This is due to the improved physical interactions between the GTR/NBR matrix and highly dispersive silica when using peroxide systems. It was confirmed using the analysis of the Wolff activity coefficient, indicating the enhanced synergy.

## 1. Introduction

Recycling rubber products is time-consuming, energy-intensive, and costly, since the material is obtained by irreversible vulcanization. The secondary compounds obtained from recycling exhibit worse properties than the originals and the raw materials, which makes them comparatively uncompetitive. The combination of the difficulties in recycling plus the properties of the obtained materials is in the origin of the economic and mainly environmental issues.

More than 80% of rubber waste is constituted by worn tires [1]. The process of the degradation of tires can take more than 100 years. The high number of worn tires and their slow degradability are features inherent to this industrial waste. It is estimated that about one billion used tires are generated each year. The accumulation of tires causes fire hazards [2] and their combustion emits toxic products that pollute rivers, lakes, and groundwater, bringing unpleasant and dangerous effects to humans and animals. In addition, the carbon dioxide released during the fire influences the greenhouse effect [3].

The most efficient approach to avoid these issues would be transforming used rubber goods into products presenting interesting properties, ideally, equivalent to those obtained with virgin rubber. To achieve this goal, the industry and academia have proposed the devulcanization of waste rubber, consisting of eliminating the cross-links created during vulcanization. With the waste rubber devulcanization, the structure of the network is broken, which enhances the processing of cross-linked rubber. The raw material, obtained by grinding the tires, is called ground tire rubber (GTR). It can be treated using physical reclaiming processes such as cryo-mechanical, microwaves, mechanical, thermo-mechanical, and ultrasonic [4,5,6].

Reclaiming is often insufficient in application issues and its effectiveness is generally associated with undesirable side-effects such as the excessive generation of hazardous volatile organic compounds, the threat of spontaneous combustion due to heating of the material, or the release of hazardous compounds into the atmosphere (unreacted, migrating components used in the manufacture of tires). One of the approaches to reduce unwanted effects and increase the application potential of waste tires is the addition of additives such as another elastomer or thermoplastic matrix, binders [7], or active fillers. Several types of compounds including other elastomeric or thermoplastics materials have been proposed and tested for different applications. Supitcha et al. [8] have used modified GTR as the base of an anion exchange resin, concluding that the ion exchange capacity of this compound could qualify it as an adequate adsorbent for defluorination. Orrit et al. [9] have used modified GTR/PVC compounds as electrical insulators, apt for spacers or electrical cables. Colom et al. [10] proposed a new compound made by modified GTR blended with waste PVC with excellent acoustical properties to be used as a sound absorber in panels for walls and ceilings. This line of action gives the waste material new functionalities. One example is nitrile rubber (NBR), which exhibits excellent oil resistance, but lacks the self-reinforcing ability and stress-induced crystallization effect [11], which can be overcome by the application of fillers.

The preparation of NBR/GTR blends allows for the tailoring of the materials with unique properties at reasonable costs. Baeta et al. [12] showed that the post-production waste of styrene-butadiene rubber (one of the main components in GTR) can be successfully used as a cost-effective, semi-reinforcement filler in the NBR matrix (a tensile strength improvement was observed up to 70 phr of waste SBR as a filler).

Moon et al. [13] and Choi et al. [14] studied the fire resistance behavior of NBR/GTR foamed composites modified with various commercially available flame retardant additives. One of the findings in the results was the fact that GTR itself is able to reduce the flammability of NBR/GTR foams.

However, due to the significant differences in the polarity of the NBR (polar) and GTR (non-polar) the research work focused on, the compatibility of such a system should be also improved. Zhang et al. [15] showed that the tensile properties in NBR/GTR systems can be improved by the plasma treatment of GTR. This is related to the enhanced interfacial interaction between the modified GTR and the NBR matrix.

Some other interesting compounds that have been developed include nitrile rubber, natural rubber, and GTR devulcanized by microwaves aiming to improve the compatibility, as in the work of Cañavate et al. [16] or the study of Zedler et al. [17] who investigated the effects of chemical additives in mechano-chemically reclaimed GTR/NBR blends.

It seems that a more suitable and cost-effective strategy to enhance matrix–filler interactions is a simple optimization of the used components, especially the type and amount of the used filler or curing system [18,19,20,21]. It should be pointed out that both of the above-mentioned additives, fillers [22,23] and curing additives [24,25], might also act as efficient compatibilizers of multiphase polymeric materials.

However, to the best of the authors’ knowledge, there are no reports in the literature about the combined impact of filler content and curing system type on GTR/NBR-based composites.

In this study, the GTR/NBR blends reinforced with the variable content of highly dispersive silica were cured using a sulfur-based system and two types of commercially available peroxide initiators (dicumyl peroxide (DCP) and di-(2-tert-butyl-peroxyisopropyl)-benzene (BIB)). The obtained materials were subjected to the process of vulcanization and a number of tests were carried out to determine the physico-mechanical, thermal, acoustic, and morphological properties of all the systems.

## 2. Materials and Methods

### 2.1. Materials

GTR obtained by ambient grinding of used tires (a combination of passenger car and truck tires in 50:50 mass ratio) with particles size below 0.8 mm, produced by Orzeł S.A. (Poniatowa, Poland) was used during this research.

NBR Ker N-29 with 27–31 wt.% of bonded acrylonitrile and Mooney viscosity ML (1 + 4), 100 °C: 45–55 was obtained from Synthos S.A. (Oświęcim, Poland). Toluene used for chemical analyses and the curing agents for vulcanization were obtained from Avantor Performance Materials Poland S.A. (Gliwice, Poland) and Standard Sp. z o.o. (Lublin, Poland).

Highly dispersive amorphous silica (Zeosil 1165 MP) with a molecular weight of 60.2 g/mol, melting point at above 1700 °C, and density 2.1 g/cm^3^ was supplied by Solvay Poland (Włocławek, Poland).

The three curing systems chemicals are (a) sulfur-based system; (b) dicumyl peroxide, and (c) di(2-tert-butyl-peroxyisopropyl) benzene.

Sulfur curing system consists of the following in phr: ZnO; 3.0, stearic acid; 2, TMTD; 1.0, and sulfur; 2.0. All ingredients in the sulfur curing system were provided by Standard Sp. z o.o. (Lublin, Poland). Organic peroxides were supplied by Pergan GmbH (Bocholt, Germany). The chemical structure and characteristics of used peroxide curing system are presented in Table 1.

### 2.2. Sample Preparation

GTR/NBR/SiO_2_ compounds were mixed with three curing systems (sulfur-based, DCP, and BIB) using a two-roll mill from Buzuluk (Komarov, Czech Republic). The formulation, displayed in Table 2, was as follows in parts per hundred of rubber (phr): 70.0 GTR; 30.0 NBR; curing system (sulfur-based, DCP, BIB) and variable content of SiO_2_ (0, 5, 15, 30 phr). The compounds were vulcanized into 2-millimeter sheets and cured at 160 (sulfur curing system) and 180 °C (peroxide curing system) using an electrically heated press PH-90 (Nysa, Poland) under the pressure of 4.9 MPa according to the optimum cure time determined as stated in ISO 3417 standard. In order to facilitate the identification of specific samples, special coding was created according to GTR/NBR_A/SiX_, where A stands for curing system, while X stands for the amount of silica.

### 2.3. Measurements

The vulcanization process of the samples was investigated via Monsanto R100S rheometer (St. Louis, MO, USA) with the oscillating rotor, following ISO 3417. To determinate the cross-linking rate, the cure rate index (*CRI*) was calculated according to Formula (1) as follows [26]:

(1) $$CRI=\frac{100}{t_{90}-t_{2}}$$

where *t*_90_: optimum vulcanization time, min; *t*_2_: scorch time, min.

The determination of the *R*_300_ parameter allowed us to investigate the thermal aging resistance of the prepared samples during curing at elevated temperature. *R*_300_ is obtained from the time at which torque reaches the maximum value (*M*_max._) and it describes the percentage of reversion degree after a period of 300 s [27]. It was calculated according to Equation (2) as follows:

(2) $$R_{300}=\frac{M_{\max.}-M_{300s}}{M_{\max.}}\times 100\%$$

where *M*_max._: maximum torque, dNm; *M*_300s_: torque 300 s after maximum torque, dNm.

The tensile strength and elongation at break were measured in accordance with ISO 37. Tensile tests were carried out on a Zwick Z020 machine (Ulm, Germany) at a constant speed of 500 mm/min. Direct extension measurements were conducted using an extensometer with sensor arms. The reported results are an average of five measurements for each sample. Shore hardness type A was assessed using a Zwick 3130 durometer (Ulm, Germany) according to ISO 7619-1.

The density was determined based on the Archimedes method, as explained in ISO 1183. Measurements were carried out at room temperature in a methanol medium, without exception.

The swelling degree of the vulcanized samples (0.2 g) was estimated via a swelling test, carried out in toluene at room temperature. The swelling degree was calculated according to Equation (3) as follows:

(3) $$Q=\frac{m_{t}-m_{o}}{m_{o}}\times 100\%$$

where *Q*: swelling degree, %; *m_t_*: a mass of the sample swollen after time t, g; and *m_o_*: an initial mass of the sample, g.

Sol fraction was calculated in accordance with Formula (4) as follows:

(4) $$Sol\;fraction=\frac{W_{1}-W_{2}}{W_{1}}\times 100\;\%$$

where *W*_1_: mass of the vulcanized sample before swelling, g; and *W*_2_: mass of the vulcanized sample after extraction, g.

According to the following Flory–Rehner Equation (5) [28], cross-link density can be determined by equilibrium swelling in toluene:

(5) $$v_{e}=\frac{-\left[\ln(1-v_{r}\right)+v_{r}+{\chi v}_{r}^{2}]}{\left[v_{1}\left(v_{r}^{1/3}-v_{r}/2\right)\right]}$$

where *ν_e_*: cross-link density, mol/cm^3^; *V_r_*: gel volume in the swollen sample; *V*_1_: solvent molar volume (toluene = 106.2, cm^3^/mol [12]); and χ: polymer–solvent interaction parameter (in the calculations, it was assumed to be 0.472 [12]).

In the case of rubber compounds modified with GTR, which includes an active filler such as carbon black, and for filled compounds, the Krause correction can be applied [29]. Actual cross-link density can be calculated with the Krause correction according to Equations (6) and (7) as follows:

(6) $$v_{\mathrm{after}\;\mathrm{correction}}=\frac{v_{e}}{1+K+\Phi }$$

(7) $$\Phi =\frac{\varphi_{f}\times \rho_{r}\times m_{0}}{\rho_{f}\times m_{dry}}$$

where *ν_e_*: the measured chemical cross-link density, mol/cm^3^; *ν_after_*_*correction*_: the actual chemical cross-link density, mol/cm^3^; K: constant characteristic of the filler but independent of the solvent; *ϕ_f_*: the volume fraction of filler in the sample that is calculated; *ρ_r_*: the density of studied compound, g/cm^3^; *m*_0_: the weight of the sample before extraction, g; *ρ_f_*: the density of filler, g/cm^3^; and *m_dry_*: the weight of the sample after extraction, g.

For this research, the density of carbon black was selected to be 1.85 g/cm^3^ and the K constant was selected to be 1.17 [30].

In order to understand the interaction between polymer matrix and silica, the reinforcing activity of the filler was assessed using Wolff activity coefficient: α_F_ [31]. Correlation between Δ*M_SPEC_* and α_*F*_ was calculated according to Equations (8) and (9) as follows:

(8) $$∆M_{SPEC}=\frac{∆M_{x}}{∆M_{0}}-1$$

(9) $$∆M_{SPEC}=\alpha_{F}\cdot ∆\frac{m_{x}}{m_{p}}$$

where Δ*M_x_*: the torque increment of the vulcanizate containing x phr of filler during vulcanization, dNm; Δ*M*_0_: the torque increment of an unfilled vulcanizate, dNm; *m_x_*: the weight of added filler, g; and *m_p_*_:_ the weight of the polymer in vulcanizate, g.

TGA studies were carried out using Mettler Toledo TGA/SDTA 851. Initially, measurements were made under nitrogen at a flow rate of 60 mL/min of gas at a heating rate of 20 °C/min. At 550 °C, airflow was introduced, and the heating rate was reduced to 10 °C/min.

ATR-FTIR spectra were acquired using an Avatar 320 spectrometer from Nicolet equipped with a CsI optical collimated and horizontal attenuated total reflectance (HATR) accessory. The internal reflecting element (IRE) was a ZnSe crystal set at an incidence angle of 45. ATR spectra were collected in the range of 4000–650 cm^−1^ by averaging 40 scans at 4 cm^−1^ of resolution. To avoid local differences in the composition of the samples produced by variations in a dispersion of the components, samples for FTIR were extracted from selected representative regions of the test specimen.

The acoustic properties had been measured using a two-microphone impedance tube Bruël and Kjaer type 4206 in the frequency range 100–6500 Hz, according to the specification ASTM E 1050, which describes the standard test method for impedance and absorption of acoustical materials.

Scanning electron microscopy (SEM) was used to qualitatively examine the fracture surface of the samples broken by the mechanical tests to study the compatibility with silica at the GTR/NBR interface. Several images of the samples were taken in a JEOL 5610 microscope. Prior to the observations, the samples were covered with a fine layer of gold-palladium in order to increase their conductivity.

## 3. Results and Discussion

### 3.1. Curing Characteristics

**Figure 1:**
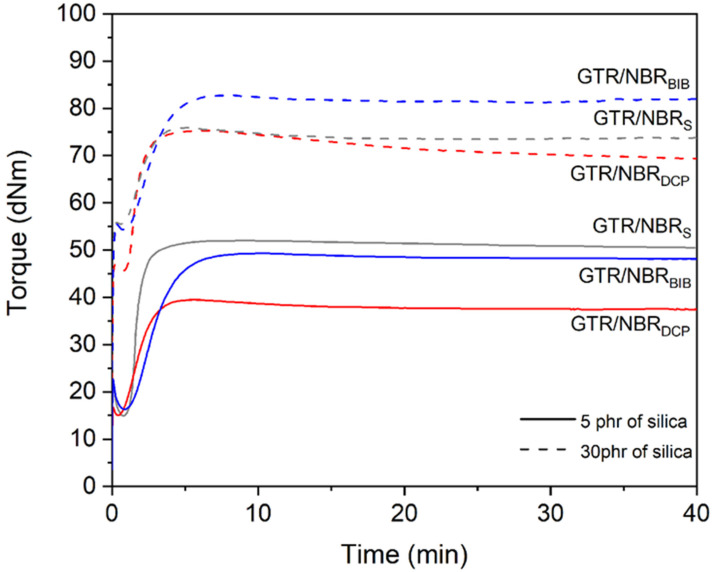
Curing curves of studied GTR/NBR performed at 160 °C (sulfur curing system) and at 180 °C (peroxides).

The effect of different curing systems on GTR/NBR/SiO_2_ compounds’ curing characteristics is presented in Figure 1 and summarized in Table 3. Two curing temperatures were used when analyzing the results, 160 °C for sulfur and 180 °C for peroxides. This is due to the nature of the systems used.

Analyzing the data, it is obvious that the minimum torque, which is a numerical value that determines the processability of the material, for the sulfur cross-linked samples increases with the increasing amount of filler (10.5, 14.9, 24.0, and 45.6 dNm for 0, 5, 15, and 30 phr loadings of highly dispersive silica, respectively), which is in accordance with many independent studies about silica-filled elastomers [32,33,34]. The same trend is observed analyzing the maximal torque values (49.8, 52.0, 59.1, and 75.3 dNm for 0, 5, 15, and 30 phr loading of silica, respectively), indicating a drastic increase in the stiffness of the tested materials. The opposite phenomenon was noted for torque increment values (39.3, 37.1, 35.1, and 29.7 dNm for 0, 5, 15, and 30 phr loading of silica, respectively). This parameter is correlated to the cross-link density of the samples. The same volume of sample is always analyzed during the oscillating disc rheometer test; therefore, as the amount of filler increases, the volume of NBR and GTR that can cross-link with the sulfur system decreases. The scorch time (1.2, 1.2, 1.0, and 1.1 min for 0, 5, 15, and 30 phr loading of silica, respectively) and the optimum cure time (2.9, 2.7, 2.4, and 2.7 min for 0, 5, 15, and 30 phr loading of silica, respectively) do not change significantly for all the samples in the series. This means that the cross-linking system used is equally effective for all the samples, and the presence of silica only affects the values related to the stiffness and processability of the material. Parameter R_300_ increases with an increasing silica content (0.3, 0.6, 1.2, and 1.8% for 0, 5, 15, and 30 phr loading of silica, respectively). A similar observation was described by Kazemi et al., who investigated natural rubber hybrid composites filled with maple/silica/carbon black [35]. The authors indicated that a high silica content (20 phr) in the studied systems resulted in an agglomeration formulation, which was related to hydrogen bonding between the silanol groups present on the silica surface.

For peroxide curing systems, the M_min._ and M_max._ values’ trend is the same as for the sulfur-cross-linked samples. Regarding the comparison of the values between the types of peroxides used, it is that the M_max._ value for the samples cross-linked with BIB peroxide is higher (38.1, 39.5, 54.6, and 75.9 dNm—DCP; 45.0, 49.3, 60.5, and 82.8 dNm—BIB). This is related to the structure of BIB peroxide [36,37], which generates a high number of free radicals compared to DCP and, as a consequence, results in a higher cross-link density and stiffness of the tested material.

It is confirmed by ∆M values, which are higher for BIB peroxide than for DCP (23.2, 24.4, 27.3, and 20.3 dNm—DCP; 31.2, 33.0, 35.0, and 28.5 dNm). It is interesting that, with 30 phr of silica, the value drops for 25.7% (DCP) and 18.6% (BIB) compared to 15 phr of the filler. A similar tendency was observed for the samples with the sulfur-based system. This phenomenon can be related to the agglomeration of silica filler in the GTR/NBR matrix when the 30 phr is used.

An analysis of the optimal vulcanization time and scorch time has shown that, despite the higher active oxygen content, the number of radicals generated, and the similar half-life temperature, BIB shows lower efficiency in terms of curing time. This is due to the higher number of radicals generated by BIB compared to DCP; therefore, for BIB, the formulation of a three-dimensional network, stabilization, and the termination of free radical takes more time.

The R_300_ parameter values of DCP cross-linked materials increase for 5 phr of silica, while it starts to decrease after this value (15 and 30 phr of silica). However, in the case of BIB, these values are constant regardless of the amount of filler used. As mentioned earlier, BIB exhibits longer curing times, making it not as rapid as DCP. A more intense free radical generation process can not only lead to cross-linking but also to elastomer chain degradation, as indicated by the higher values of the R_300_ parameter. Moreover, with the increasing content of the filler, the values shift towards 0 value, which corresponds to better resistance for reversion during curing. This phenomenon can be related to the partial devulcanization of GTR. It is well known that the efficiency of rubber devulcanization is strongly affected by temperature [38,39], and usually increases with a higher temperature (in the studied case, 180 °C for peroxide curing system and 160 °C for curing system temperature). The partial devulcanization of GTR has a significant impact on the interfacial interaction in matrix–GTR. This is due to the additional physical interactions and also the possible migration of components between matrix and GTR, which was comprehensively described in work [40].

High temperature and pressure also enhanced the sintering of GTR particles without using additional additives [41,42]. This is related to the combined effects of the devulcanization, degradation, and secondary vulcanization (revulcanization) of GTR (or GTR with NBR) supported by free radicals’ reactions.

Moreover, it should be pointed that during peroxide curing, it is also possible that the peroxide-induced degradation of GTR might occur. Sabzekar et al. [43] and Colom et al. [44] studied the effect of BPO on the thermo-mechanical devulcanization of waste rubber, but due to a similarity in the peroxide decomposition mechanisms for other peroxides, similar effects can be expected. In the case of BIB and DCP, some differences are related to their affinity of GTR and NBR.

Furthermore, it should also be pointed out that the components also present in GTR (e.g., carbon black, curing agents’ residue, etc.) might have an impact on the efficiency sulfur curing system [45,46] and peroxides [47,48] during the formulation of materials modified with GTR.

**Figure 2:**
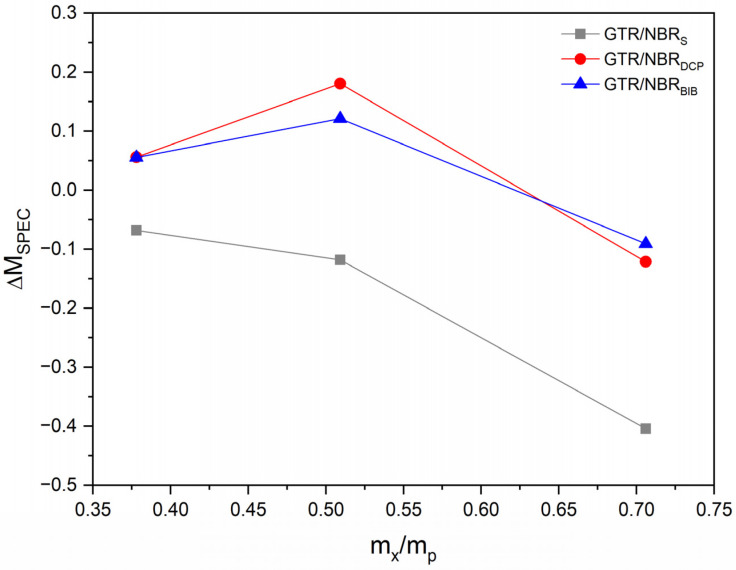
Wolff activity coefficient as a function of silica content for all applied systems.

As already noted, the amount of filler has a critical effect on the cross-linking characteristics of the sample. The vulcanization mechanism is affected mainly by two factors, first, the physical inhibition caused by the silica, which reduces the mobility of the rubber chains and prevents part of the rubber from participating in the vulcanization. This phenomenon is also present during the curing with sulfur. Second, the effect of the hydroxyl surface groups of the silica, which promotes the radical formation, but also enhances the agglomeration of silica. Depending on the amount of silica and curing system type, and the competition of physical and radical promotion effects, vulcanization can be affected greatly. The obtained results (changes of ∆M parameter) are confirmed by the calculation of the Wolff activity coefficient as presented in Figure 2. Interaction decreases for the sulfur system, while for peroxides, it slightly rises and then drops for 30 phr. This phenomenon can be related to the possible migration of carbon black from partially devulcanized GTR to the NBR matrix during the processing of the studied materials [49,50]. It should be highlighted that during the calculation of the Wolff activity coefficient, the carbon black present in GTR was not included as a filler phase (assumption based on the fact that the content of carbon black in GTR is constant).

The second one shows better behavior and this is reflected in the strength of the material. The results may be due to restricted SiO_2_ diffusion due to the high cross-link density, as well as the vulcanization kinetics of a peroxide-based system by the presence of silica. When the amount of silica increases to 30 phr, the interaction decreases because the amount of SiO_2_ is too high, and agglomerates can be formed.

### 3.2. Physico-Mechanical Properties of GTR/NBR/SiO_2_

The physico-mechanical properties of the samples are shown in Table 4. The analysis of the results, for the sulfur system, shows an increase in tensile strength (5.2 ± 0.7, 6.1 ± 0.6, 7.5 ± 0.9, and 8.5 ± 1.5 MPa), modulus at 100% (3.2 ± 0.9, 3.6 ± 0.8, 4.6 ± 0.6, and 6.8 ± 0.9 MPa), and hardness (69 ± 1, 69 ± 1, 74 ± 2, and 81 ± 2 ShA) as the amount of filler increases, while elongation at break decreases (168 ± 17, 167 ± 19, 162 ± 20, and 131 ± 21%). Those results simply show that an increasing amount of silica rises the stiffness of the material resulting in higher tensile strength, modulus at 100%, and hardness, while limiting the flexibility and rotation of the elastomer chains (decrease in elongation at break). Those assumptions are in accordance with M_max._, ∆M, and the Wolff activity coefficient.

Interesting relationships were observed when peroxide systems were used. In terms of tensile strength, modulus at 100%, and hardness, the increasing trend was maintained. However, in terms of elongation at break, the parameter behaves according to the value of ∆M and the Wolff activity coefficient (increase up to 15 phr of silica, then the value drops), indicating the enhancement and physical interactions occurred between the elastomeric matrix and silica due to the application of peroxides and the higher temperature during curing. As mentioned in the previous section, this observation can be related to the following three factors: (i) the partial devulcanization or revulcanization of GTR during the compression of composites at 180 °C; (ii) the possible migration of carbon black from the GTR phase to NBR; (iii) the tendency of silica filler to agglomerate.

There are also important differences based on the type of peroxide used. DCP has a lower tensile strength (5.1 ± 0.3, 5.6 ± 0.3, 7.2 ± 0.6, and 10.0 ± 1.0 MPa), modulus at 100% (2.0 ± 0.8, 1.9 ± 0.3, 2.6 ± 0.7, and 4.3 ± 0.8 MPa), and hardness (54 ± 2.57 ± 1.65 ± 1, and 78 ± 1 ShA) compared to BIB (6.0 ± 0.4, 6.8 ± 0.5, 8.4 ± 0.5, and 10.2 ± 0.6 MPa; 2.4 ± 0.3, 2.5 ± 0.5, 3.1 ± 0.8, and 5.0 ± 0.6 MPa; 58 ± 1.61 ± 1.68 ± 1, and 81 ± 1 ShA, respectively, for tensile strength, modulus at 100%, and hardness). The higher values are due to the higher stiffness of the material, which, in turn, is due to the amount of generated free radicals cross-linking the system of studied samples. In the case of elongation at break, the higher values were obtained for DCP (up to 15 phr), which only confirms the lower efficiency of the peroxide. It is interesting that the value (240 ± 23%) for 30 phr of silica is very similar to the BIB elongation at break value (225 ± 23%).

**Figure 3:**
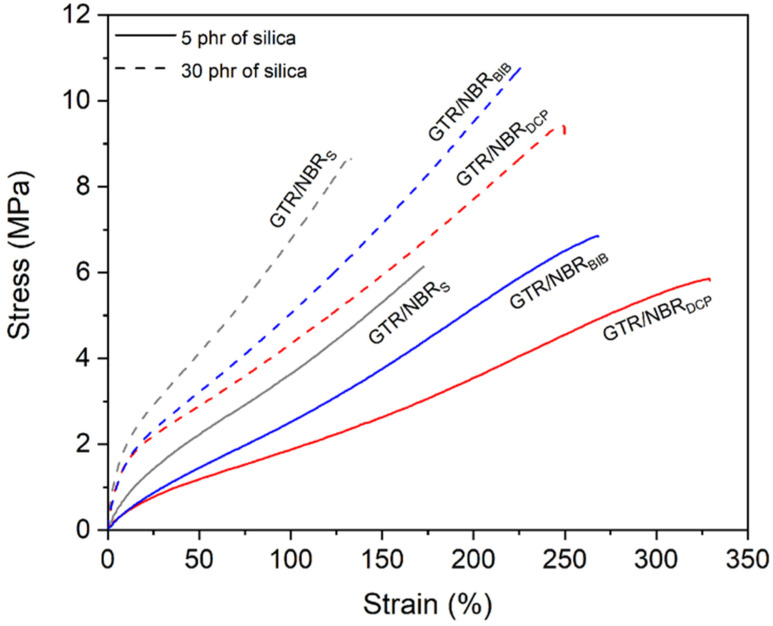
Stress–strain curves of modified GTR/NBR sintered at 160 °C (sulfur curing system) and at 180 °C (peroxides).

Figure 3 shows graphically the differences in the strain–stress curves. The courses of the stress–strain curves for the three curing systems are similar in shape. They show only the differences discussed above in terms of modulus and elongation values. This is related to similar behavior in the development of effective interactions between the matrix and filler, supported by the formulated three-dimensional network.

**Figure 4:**
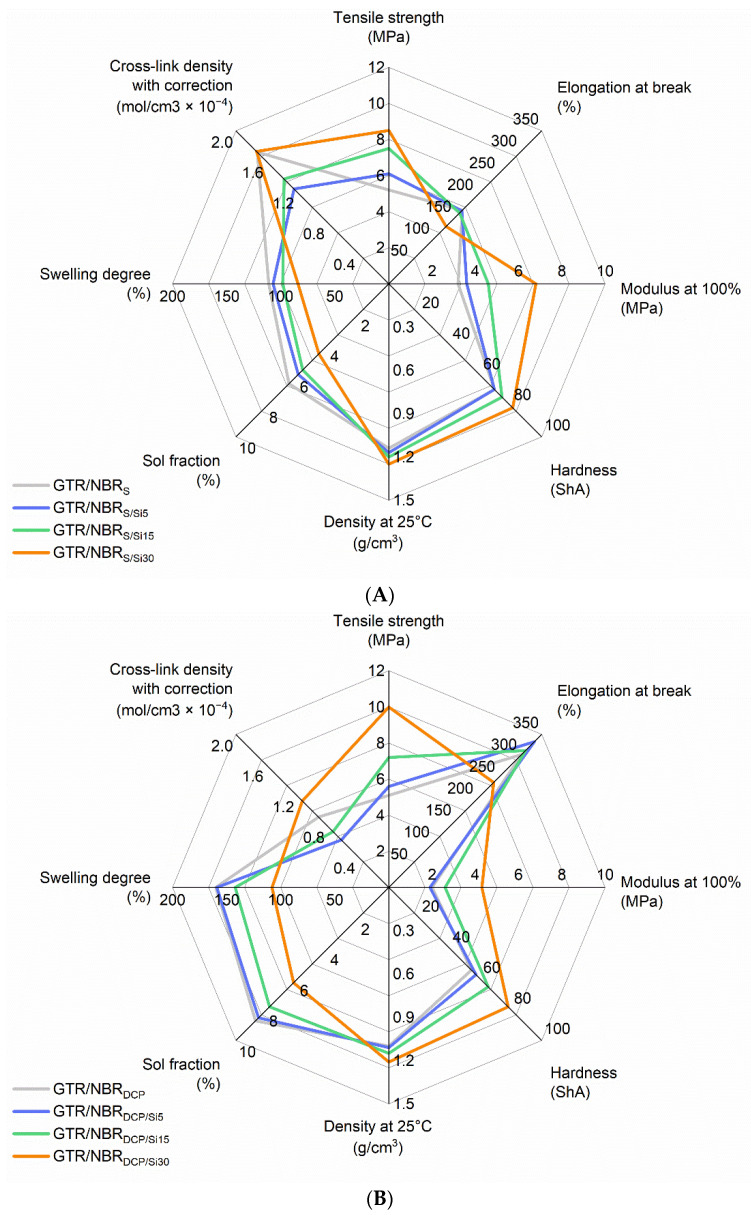

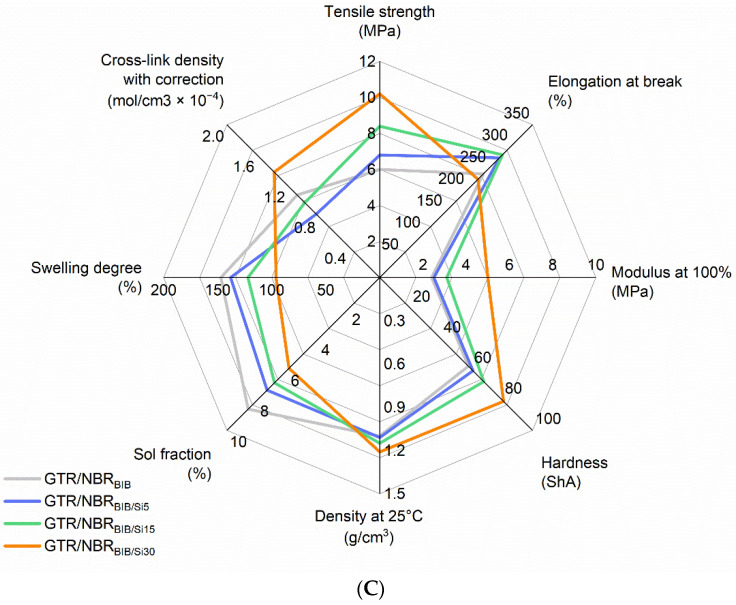
Spider chart representing the change of physico-mechanical properties of GTR/NBR samples in the function of highly dispersive silica content: (**A**) sulfur, (**B**) DCP, (**C**) BIB.

In order to further analyze the data and more easily determine the correlations between the results obtained as a function of the type of system and amount of filler, spider charts were prepared and are presented in Figure 4. The charts allow the observation of the coherence of the values obtained for the different properties, taking into account the previous discussions related to the cross-linking, microstructure, and their relationship with the tests performed on the samples. The general trend of a determined type of curing system is reflected in a deviation of the scheme towards one of the axes.

In the case of density, sol fraction, swelling degree, and cross-link density, all the samples show an identical trend in the function of silica content. The density and cross-link density increase, while the sol fraction and swelling degree decrease with an increasing content of silica. What is interesting is that the shapes of the figures obtained from plotting the analyzed data on the charts are very close to each other when peroxides are used, which confirms the identical cross-linking pattern of the compounds. In the case of sulfur, the chart has several key differences, particularly in terms of strength parameters (as discussed in the previous section of this subsection), sol fraction, swelling degree, and cross-link density. From the data obtained, it can be seen that sulfur is the most effective system studied (lower degree of swelling, lower sol fraction, and higher value of cross-link density); however, the sulfur-cured composites showed the lower mechanical properties (e.g., tensile strength) compared to composites cured by peroxides. It can be explained by the interaction between GTR, NBR, and the silica phase, supported by peroxide curing at a high temperature, hence, the enhanced interactions between the used components (as the combined effects of GTR devulcanization and silica dispersion level in the GTR/NBR system). This shows that the effect of reinforcement by highly dispersed silica is more important in terms of physico-mechanical properties than the degree of cross-linking of the sample.

### 3.3. FTIR Analysis

**Figure 5:**
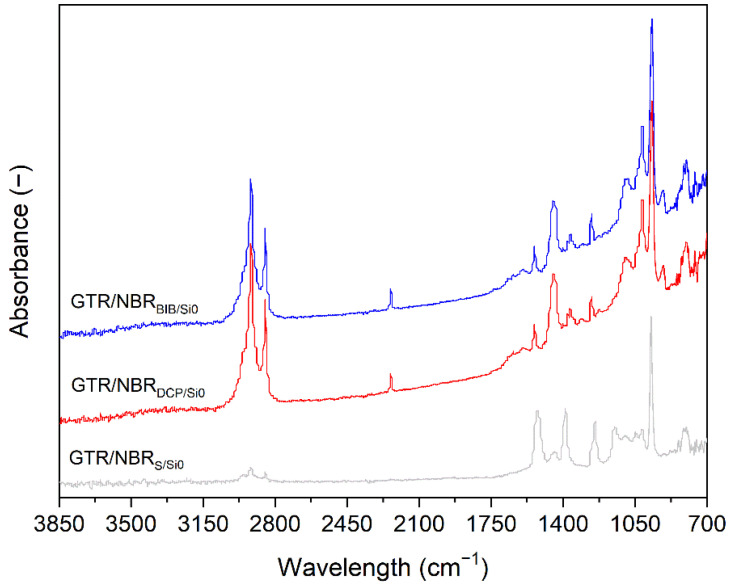
FTIR spectra of GTR/NBR blends without silica.

**Figure 6:**
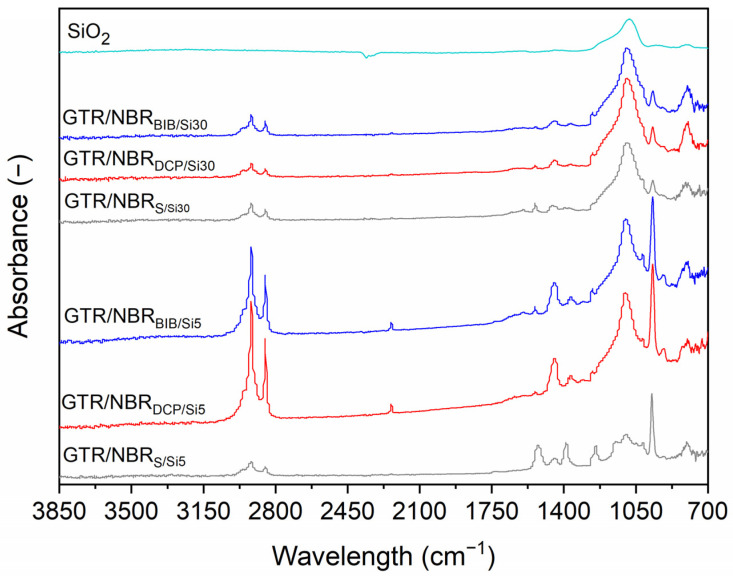
FTIR spectra of GTR/NBR blends filled with 5 and 30 phr of silica.

Figure 5 and Figure 6 show the spectra of GTR/NBR compounds without and with silica cured with the three systems studied in the 700–3850 cm^−1^ range. The absorbance maxima in 2965 cm^−1^ is related to the aromatic C-H bonds vibrations (e.g., styrene-butadiene rubber and carbon black present in GTR). The signals in the rank of 2850–2950 cm^−1^ are attributed to the C-H vibrations of CH_2_ groups present in the structure of elastomers. As can be observed, the intensity of these signals for composites cured with peroxides are much higher compared to sulfur-cured samples (the same tendency was observed for samples with 5 phr of silica, see Figure 6). This can be due to the following two factors: (i) during the decomposition of peroxides, low molecular by-products are formulated, and some residual is left on the material surface; (ii) in the GTR/NBR blends, a possible migration of the carbon black (strong absorber of infrared radiation [51]) present in GTR occurs and its efficiency is correlated with the progress of GTR devulcanization. Regardless of the curing system type, the level of the signal in 2850–2950 cm^−1^ is reduced with the increasing content of silica.

The sample cured with sulfur presents two significant FTIR bands at 1538 and 1398 cm^−1^ assigned to zinc stearate formed during the reaction between ZnO and stearic acid during rubber compounding [6]. These bands are only present in the samples cured with the sulfur curing system because samples with DCP or BIB do not include this component. The bands at 1260 and 1150 cm^−1^, also specific to the sulfur samples, are assigned to C-O products obtained in reactions of sulfur systems.

Other relevant bands, common to the presented spectra are 1450 cm^−1^ related to the CH_2_− stretching GTR/NBR bands; a small band at 1375 cm^−1^, corresponding to the CH_3_ symmetric bend of GTR; a small band at 1018 cm^−1^ related to the C-C of carbon black contained in GTR and 964 cm^−1^ (trans) RCH=CHR’.

As reported in a previous work [52], there is a weak FTIR signal at 1540 cm^−1^, which is present in the samples cured with BIB and DCP. This is attributed to the first vulcanization of GTR, which has been made using the sulfur system and, therefore, also contains some ZnO and stearic acid.

FTIR spectra of GTR/NBR samples filled with 5 and 30 phr of silica and the spectrum of SiO_2_ are presented in Figure 6. In this figure, the increase in the band present in the SiO_2_ spectrum is comparatively evident in the studied samples according to their composition. A wideband at 1090 cm^−1^ increases with the content of SiO_2_ overlapping other bands. There is also a comparative diminution of the intensity of all the bands with the increase in SiO_2_, observed especially at the contents of SiO_2_ around 30 phr. These spectra were obtained using ATR, which measures the surface of the samples; the results are indicative of a migration of the particles of SiO_2_ from the core of the sample to the surface.

In our previous work [52], we also observed that the materials cured with the sulfur system present a higher cross-link density than the ones cured with DCP and that produces a lower signal in the ATR spectroscopy of NR/GTR. This phenomenon is also produced here. The samples cured using the sulfur process present less migration from the core to the surface of the SiO_2_ and this is observed as a smaller increase in the related band. The data are coherent with the previous results in cross-linking.

FTIR analysis demonstrated that the silica filler and type of curing system used have a significant influence on the chemical structure of the obtained GTR/NBR-based composites, which is due to the difference in the three-dimensional network formulated by the sulfur or peroxide system and also the level of physical interactions between GTR/NBR and silica filler supported by GTR devulcanization.

### 3.4. Thermogravimetric Analysis

**Figure 7:**
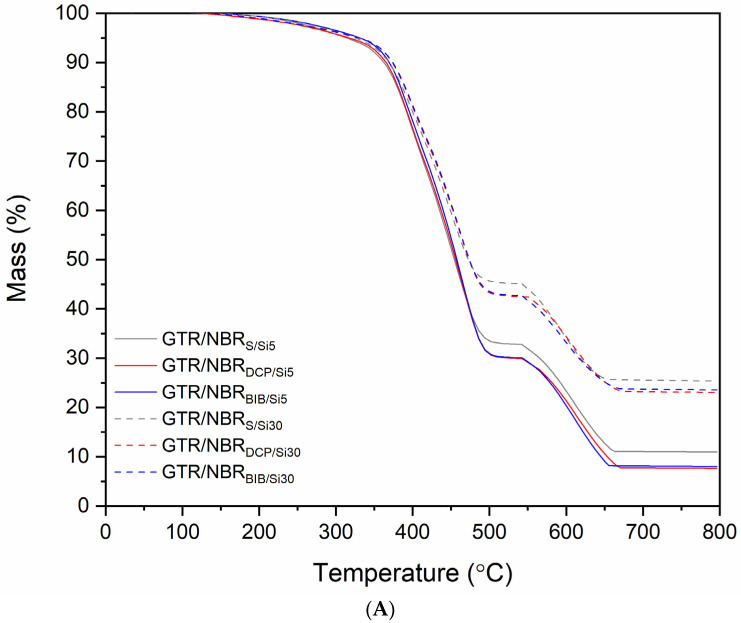

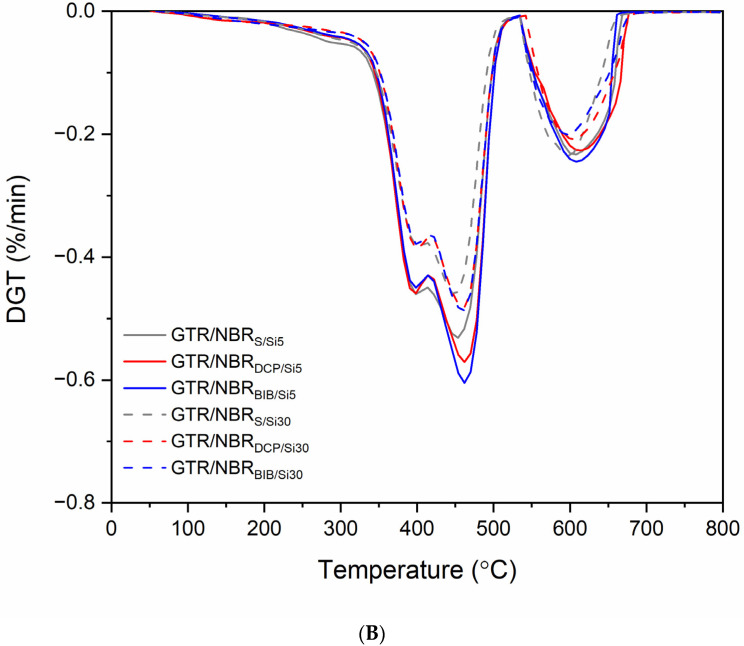
(**A**) TGA curves and (**B**) DTG results for GTR/NBR samples filled with 5 and 30 phr of silica.

The results of the thermogravimetric analysis of GTR/NBR/SiO_2_ samples are presented in Figure 7 and summarized in Table 5. It was observed that the type of curing system and the amount of SiO_2_ influence TGA thermograms, resulting in a different thermogram for every sample studied. This is coherent with the results presented previously. To avoid the combined effect of thermal and thermo-oxidative degradation, initially, the measurements were made under nitrogen, although to compile the thermo-oxidation of the samples at 550 °C, airflow was used. This experiment was carried out until the degradation of the organic components and carbon black was complete, to determine the stability of the composites and the number of inorganic residua (mainly SiO_2_ and other compounds generated in the thermo-oxidation process). Different data have been obtained at 2, 5, 10, and 50% of weight loss, which corresponded to the T_−2%_, T_−5%_, T_−10%,_ and T_−50%_ temperatures. The values corresponding to the 2% were higher for the sulfur curing system than in the samples cured by peroxides. This is due to the nature of cross-linking in the presence of peroxides. As a result of the generated free radicals, not only crosslinking but also the scission of the main bonds can occur, resulting in the formation of low molecular weight compounds. Moreover, it should be mentioned that during the decomposition of peroxides, low molecular by-products are also formulated. These compounds generally have a lower degradation temperature than the rubber itself, and for this reason, a faster onset of degradation was observed for the peroxides.

Figure 7 shows the thermograms (TGA and DTG) for all the samples. The increase in the char residue of the samples is due to the presence of a high proportion of silica in the composites. In sulfur-cured samples, some inorganic compounds generated by reaction in the thermo-oxidative process also appear. It is also worth remarking that the DTG plot shows that thermal stability increases with silica and it is slightly higher in samples cured with peroxides. In these DTG curves, three peaks are observed, assigned to (a) the thermal decomposition of the NR and BR of GTR at 410 °C, (b) the thermal decomposition of the SBR of GTR at 465 °C and NBR at 470 °C, and (c) a broad peak at 630 °C that corresponds to the thermal decomposition of carbon black [53].

### 3.5. Acoustic Properties

**Figure 8:**
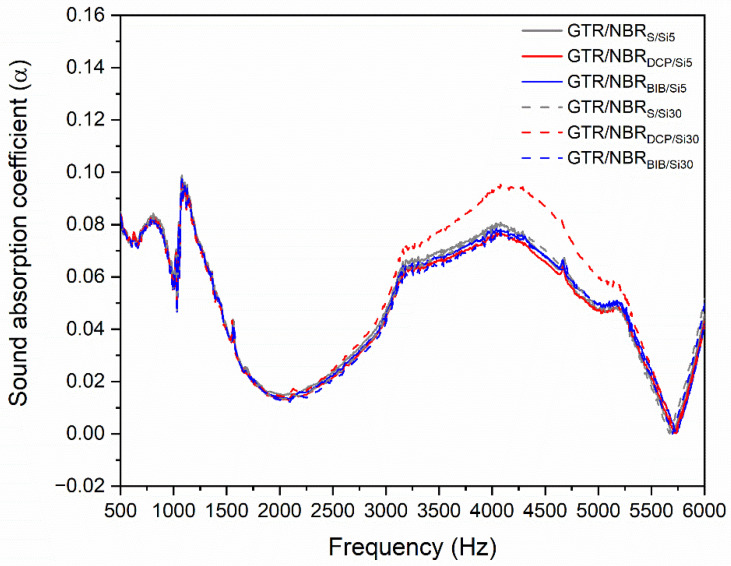
Sound absorption coefficient as a function of frequency for GTR/NBR samples.

The sound absorption coefficient as a function of the frequency of the studied samples is presented in Figure 8. All the materials presented showed similar curves, regardless of the amount of filler and the type of cross-linking system. The only noticeable difference is the peak height in the 3000–5000 Hz range for sample GTR/NBR_DCP/Si30_. The greatest influence on the acoustic properties of materials is their physical and chemical structure, as well as being strongly related to the density of the test samples [54].

Therefore, the acoustic properties result directly from the type of material, particle size distribution, methods of its preparation, and thickness [55]. Taking that into consideration, and the fact that all the samples were characterized with a relatively high degree of cross-linking, one may conclude that is the reason for the similar sound absorption behavior.

The sound absorption is related to sample structure, properties, thickness, and surface conditions, as well as to the incident angle and frequency of the sound waves [56].

During the test, analysis is performed over a wide range of frequencies (500–6000 Hz), and the values change depending on the frequency value. Therefore, the sound-absorbing property can be determined by analyzing specific frequency values (500, 1000, 2000, and 4000 Hz). When the average frequency equals or is higher than 0.2, the tested material can be called “sound-absorbing”. In Table 6, the sound absorption coefficient for the selected regions and its average value is presented. The results show that none of the tested materials are good enough to be a sound-absorbing material.

### 3.6. SEM

**Figure 9:**
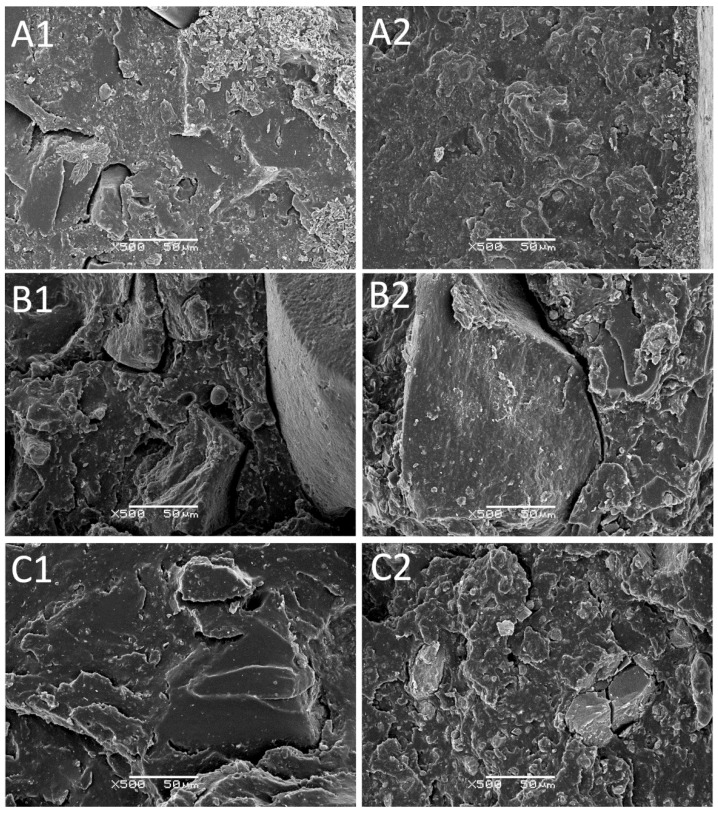
SEM images of samples: (**A1**) GTR/NBR_S/Si5_, (**A2**) GTR/NBR_S/Si30_, (**B1**) GTR/NBR_DCP/Si5_, (**B2**) GTR/NBR_DCP/Si30_, (**C1**) GTR/NBR_BIB/Si5_, (**C2**) GTR/NBR_BIB/Si30_ (magnification ×500).

SEM micrographs (magnification ×500) of tensile fractured samples are presented in Figure 9. Figure 9A includes the GTR/NBR compounds cured by sulfur with 5 and 30 phr silica, Figure 9B analog compounds cured with DCP, and Figure 9C with BIB. The structure of the samples in Figure 9B, corresponding to the samples cured with DCP, stands out because of its rough surface with many gaps and voids. The pictures labeled as 9C corresponding to the samples cured with BIB also present a rough surface, though they are not as notorious as the samples in Figure 9B.

The differences observed in the structure of the presented samples can be related to several factors. One of them is the curing temperature used in the preparation of the samples combined with the higher cross-link found in the samples cured with sulfur. The sulfur system is cured at 160 °C and peroxides are cured at 180 °C. These samples, as discussed above, differ in mechanical properties, and the samples cured with sulfur show a more fragile fracture than the samples leading to a cleaner surface, while in the samples cured with peroxides, the surface appears more strained and plastically deformed.

As discussed in the curing characteristics section, the higher temperature used during peroxide curing enhanced interfacial adhesion in the GTR/NBR system reinforced by silica, due to the combined effects of the devulcanization, degradation, and secondary vulcanization (revulcanization) of GTR (or GTR with NBR) supported by free radicals’ reactions. These observations corresponded with tensile properties measurements, which were higher for the composites cured by peroxides than the sulfur-based system.

Moreover, the tendency of silica to agglomerate also affects the interfacial compatibility in the studied materials. De et al. [57] investigated the impact of reclaimed rubber (GTR with a particle size up to 0.1 mm was used) on silica reinforcement (content: 11.6–16.9 wt.%) of SBR/reclaimed blends (ratio from 80/20 to 40/60 wt.%). The SEM micrographs (prepared in the same magnification as in the present study) showed that silica filler disperses uniformly in both phases: SBR and reclaimed rubber. In the present study, the GTR particles are much bigger compared to the images presented by De et al. [57], which confirms only the partial devulcanization of the GTR used (with an average particle size below 0.8 mm). Comparing the mechanical properties, De et al. [57] showed that for unfilled SBR/reclaimed vulcanizates, the tensile strength and elongation at break were 2.8 MPa and 377% for SBR/reclaimed rubber in a ratio 80/20 wt.%, while for SBR/reclaimed rubber, the ratio values for these parameters were 5.0 MPa and 455%, respectively. This confirms the possible migration of carbon black from reclaimed rubber to fresh rubber matrix. In this study, regardless of the curing system type used, for the unfilled GTR/NBR system (70/30 wt.%), the tensile strength and elongation at break were in the range of 5.1–6.0 MPa and 168–304%.

The authors [57] also indicate that for SBR/reclaimed rubber in a ratio of 40/60 wt.% filled with 11.6 wt.%, the tensile strength, elongation at break, and hardness parameters were 8.1 MPa, 403%, and 68 ShA. For comparison, in the present study, regardless of the curing system type used, the values of tensile strength, elongation at break, and hardness of the studied GTR/NBR system samples (70/30 wt.%) with 15 phr of silica (11.9 wt.% for sulfur-based system and 12.8 wt.% for peroxide system, see Table 2) were in the range of 7.2–8.4 MPa, 162–313%, and 65–74 ShA, respectively. The presented results confirmed the significant improvement of tensile properties by the incorporation of silica into the rubber compound modified by ground tire rubber, which fully justifies the future development of research in this field.

## 4. Conclusions

The study presented a complex analysis of GTR/NBR blends reinforced with different amounts of silica and cured with three various curing systems. The curing characteristic was served to give detailed information about the influence of the applied variables. Regarding the amount of silica used, deviations from the linearity of results (decrease in ΔM and Wolff coefficient) were observed after exceeding the value of 15 phr. In the case of a decreasing ΔM and Wolff coefficient, one can also expect a decrease in the mechanical properties; however, a constant increase in tensile strength with an increasing filler amount was observed. This phenomenon is due to the partial devulcanization of GTR, which enhances the interfacial interactions between the components used in the GTR/NBR/silica system. The curing characteristics indicate that the most efficient curing system is the sulfur-based one; however, the opposite outcome can be read from physico-the mechanical properties (higher tensile strength and elongation at break for peroxides). It indicates the occurrence of an enhanced interaction between GTR, NBR, and silica supported by the peroxides. These data are also in accordance with the FTIR spectra, which show a significant difference in the chemical structure of the three-dimensional network, as well as in the level of physical interactions. The thermogravimetric analysis reflects the mentioned results. Peroxides may partially degrade the structure of blends resulting in the generation of VOCs shifting T_−2%_ toward lower values. It indicates a potential risk of hazardous substances being transferred to the environment with a harmful effect on human life. This aspect requires further detailed analysis with a focus on VOCs’ detection and classification. The analysis of acoustic properties allowed us to determine the suitability of the obtained materials as sound absorbing products. For this purpose, the sound absorption coefficient was studied for four different frequencies and the average was determined. When the value is ≥0.2, the material is considered to be sound absorbing. However, the results indicate that none of the materials obtained meet the expected minimum, with values oscillating around 0.06. This means that in the case of the analyzed samples, the main factor affecting the discussed parameter is the matrix material. Despite the use of different cross-linking systems, which were characterized by different degrees of cross-linking (sulfur vs. peroxides), the coefficient remained the same. Moreover, this conclusion is supported by SEM analysis, showing differences in the morphology of the samples when different cross-linking systems and filler amounts were used.

## Figures and Tables

**Table 1:** Characteristics of used organic peroxides.

Name	Abbreviation	Chemical Structure	Active Oxygen (%) *	The Half-Life Temperature (°C) *
di-(2-tert-butyl-peroxyisopropyl)-benzene	BIB	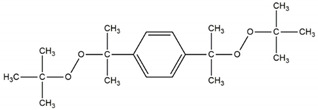	8.98	169
dicumyl peroxide	DCP	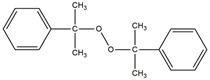	5.80	162

* Datasheet from Pergan GmbH.

**Table 2:** The composition and coding of studied samples.

Components (phr)	Sample Code											
	GTR/ NBR S	GTR/ NBR S/Si5	GTR/ NBR S/Si15	GTR/ NBR S/Si30	GTR/ NBR DCP	GTR/ NBR DCP/Si5	GTR/ NBR DCP/Si15	GTR/ NBR DCP/Si30	GTR/ NBR BIB	GTR/ NBR BIB/Si5	GTR/ NBR BIB/Si15	GTR/ NBR BIB/Si30
GTR *	70	70	70	70	70	70	70	70	70	70	70	70
NBR	30	30	30	30	30	30	30	30	30	30	30	30
Silica	-	5	15	30	-	5	15	30	-	5	15	30
Zinc Oxide	5	5	5	5	-	-	-	-	-	-	-	-
Stearic Acid	3	3	3	3	-	-	-	-	-	-	-	-
Sulfur	2	2	2	2	-	-	-	-	-	-	-	-
TMTD	1	1	1	1	-	-	-	-	-	-	-	-
DCP	-	-	-	-	2	2	2	2	-	-	-	-
BIB	-	-	-	-	-	-	-	-	2	2	2	2

* To improve processing GTR + 10 phr Modbit 25/55–60 were mixed using a two-roll mill for 10 min at ambient temperature.

**Table 3:** Curing characteristics of GTR/NBR composites as a function of curing system and silica content.

Sample Code	Curing System	Curing Temp (°C)	Silica Content (phr)	Curing Parameters							
				M_min._ (dNm)	M_max._ (dNm)	ΔM (dNm)	ΔM_SPEC_ (-)	t_2_ (min)	t_90_ (min)	CRI (min^−1^)	R_300_ (%)
GTR/NBR_S_	Sulfur based	160	0	10.5	49.8	39.3	-	1.2	2.9	57.1	0.3
GTR/NBR_S/Si5_			5	14.9	52.0	37.1	−0.07	1.2	2.7	68.0	0.6
GTR/NBR_S/Si15_			15	24.0	59.1	35.1	−0.12	1.0	2.4	75.2	1.2
GTR/NBR_S/Si30_			30	45.6	75.3	29.7	−0.40	1.1	2.7	62.1	1.8
GTR/NBR_DCP_	DCP	180	0	14.9	38.1	23.2	-	1.1	3.4	43.1	2.6
GTR/NBR_DCP/Si5_			5	15.1	39.5	24.4	0.06	0.9	3.2	43.1	3.9
GTR/NBR_DCP/Si15_			15	27.3	54.6	27.3	0.18	1.0	3.6	39.2	2.3
GTR/NBR_DCP/Si30_			30	55.6	75.9	20.3	−0.12	1.2	3.3	47.4	1.8
GTR/NBR_BIB_	BIB	180	0	13.8	45.0	31.2	-	1.3	4.8	28.8	1.0
GTR/NBR_BIB/Si5_			5	16.3	49.3	33.0	0.06	1.5	5.1	27.5	1.0
GTR/NBR_BIB/Si15_			15	25.4	60.5	35.0	0.12	1.1	4.5	29.6	1.1
GTR/NBR_BIB/Si30_			30	54.3	82.8	28.5	−0.09	1.4	4.6	31.3	1.1

**Table 4:** Physico-mechanical parameters of GTR/NBR composites as function of curing system and silica content.

Sample code	Curing System	Curing Temp (°C)	Silica Content (phr)	Physico-Mechanical Parameters							
				Tensile Strength (MPa)	Elongation at Break (%)	Modulus at 100% (MPa)	Hardness (ShA)	Density at 25 °C (g/cm^3^)	Swelling Degree (%)	Cross-Link Density with Correction (mol/cm^3^ × 10^−4^)	Sol Fraction (%)
GTR/NBR_S_	Sulfur based	160	0	5.2 ± 0.7	168 ± 17	3.2 ± 0.9	69 ± 1	1.14 ± 0.01	111 ± 1	1.72 ± 0.01	6.5 ± 0.1
GTR/NBR_S/Si5_			5	6.1 ± 0.6	167 ± 19	3.6 ± 0.8	69 ± 1	1.17 ± 0.01	107 ± 1	1.24 ± 0.01	5.9 ± 0.1
GTR/NBR_S/Si15_			15	7.5 ± 0.9	162 ± 20	4.6 ± 0.6	74 ± 2	1.20 ± 0.01	98 ± 1	1.37 ± 0.01	5.6 ± 0.1
GTR/NBR_S/Si30_			30	8.5 ± 1.5	131 ± 21	6.8 ± 0.9	81 ± 2	1.25 ± 0.01	84 ± 1	1.73 ± 0.05	4.6 ± 0.2
GTR/NBR_DCP_	DCP	180	0	5.1 ± 0.3	304 ± 12	2.0 ± 0.8	54 ± 2	1.10 ± 0.01	160 ± 1	0.92 ± 0.02	8.7 ± 0.1
GTR/NBR_DCP/Si5_			5	5.6 ± 0.3	333 ± 23	1.9 ± 0.3	57 ± 1	1.11 ± 0.01	159 ± 4	0.62 ± 0.03	8.5 ± 0.3
GTR/NBR_DCP/Si15_			15	7.2 ± 0.6	313 ± 7	2.6 ± 0.7	65 ± 1	1.15 ± 0.01	142 ± 4	0.73 ± 0.04	7.8 ± 0.2
GTR/NBR_DCP/Si30_			30	10.0 ± 1.0	240 ± 23	4.3 ± 0.8	78 ± 1	1.21 ± 0.01	108 ± 3	1.13 ± 0.07	6.2 ± 0.3
GTR/NBR_BIB_	BIB	180	0	6.0 ± 0.4	237 ± 23	2.4 ± 0.3	58 ± 1	1.10 ± 0.01	147 ± 3	1.08 ± 0.05	8.6 ± 0.3
GTR/NBR_BIB/Si5_			5	6.8 ± 0.5	274 ± 12	2.5 ± 0.5	61 ± 1	1.11 ± 0.01	138 ± 1	0.83 ± 0.01	7.4 ± 0.2
GTR/NBR_BIB/Si15_			15	8.4 ± 0.5	281 ± 5	3.1 ± 0.8	68 ± 1	1.15 ± 0.01	122 ± 1	0.98 ± 0.01	6.9 ± 0.1
GTR/NBR_BIB/Si30_			30	10.2 ± 0.6	225 ± 25	5.0 ± 0.6	81 ± 1	1.21 ± 0.01	96 ± 3	1.38 ± 0.09	5.9 ± 0.3

**Table 5:** Thermal decomposition characteristics of studied materials.

Sample	Mass Loss (%)				Char Residue at 750 °C (%)
	T_−2%_	T_−5%_	T_−10%_	T_−50%_	
	Temperature (°C)				
GTR/NBR_S/Si5_	253.2	317.5	366.2	453.6	11.0
GTR/NBR_DCP/Si5_	237.2	317.5	366.3	453.8	7.7
GTR/NBR_BIB/Si5_	237.6	333.7	366.4	457.4	8.0
GTR/NBR_S/Si30_	253.6	325.9	366.4	470.2	25.4
GTR/NBR_DCP/Si30_	237.0	325.5	374.4	469.6	23.1
GTR/NBR_BIB/Si30_	237.4	325.8	374.6	473.9	23.6

**Table 6:** The changes in the sound absorption coefficient for the studied samples at 500, 1000, 2000, and 4000 Hz.

Frequency (Hz)	Sample Code					
	GTR/NBR S/Si5	GTR/NBR DCP/Si5	GTR/NBR BIB/Si5	GTR/NBR S/Si30	GTR/NBR DCP/Si30	GTR/NBR BIB/Si30
	Sound Absorption Coefficient (α)					
500	0.08405	0.08376	0.08147	0.08266	0.08330	0.08282
1000	0.06136	0.05938	0.06085	0.06012	0.05929	0.05840
2000	0.01491	0.01344	0.01402	0.01360	0.01439	0.01292
4000	0.07988	0.07610	0.07809	0.07910	0.09323	0.07543
Average	0.06005	0.05817	0.05861	0.05887	0.06255	0.05739

## Data Availability

Not applicable.
